# Association of age and night flight duration with sleep disorders among Chinese airline pilots

**DOI:** 10.3389/fpubh.2023.1217005

**Published:** 2023-09-07

**Authors:** Ruizi Shi, Fang Wang, Wanying Xu, Li Fu

**Affiliations:** ^1^Shanghai Institute of Aviation Medicine, Ruijin Hospital Affiliated to School of Medicine, Shanghai Jiao Tong University, Shanghai, China; ^2^Shanghai Hospital of Civil Aviation Administration of China, Gubei Branch of Ruijin Hospital Affiliated to School of Medicine, Shanghai Jiaotong University, Shanghai, China

**Keywords:** age, night flight duration, interaction, sleep disorders, PSQI

## Abstract

**Objective:**

Night flights might aggravate sleep disorders among aging airline pilots, posing a threat to flight safety. In this study, we assess the prevalence of sleep disorders as well as the combined effects of night flight duration and aging on sleep disorders.

**Method:**

A cross-sectional study was conducted between July and December, 2021. Participants were recruited from a commercial airline. Sleep disorders were evaluated using the Pittsburgh Sleep Quality Index (PSQI). The interaction effect of night flight duration and age on sleep disorders and their correlates were examined using logistic regression models.

**Results:**

In total, 1,208 male airline pilots were included in the study, with a median age of 34 (interquartile range [IQR]: 29–39) years. The overall prevalence of sleep disorders was 42.6%. The multivariate logistic regression identified an interaction between night flight duration and age on sleep disorders (adjusted odds ratio [aOR] of the interaction term was 5.85 95% CI: 2.23–15.34 for age ≥ 45 years; 1.96 95% CI:1.01–3.81 for the age group 30–44 years). Longer night flight duration (aOR: 4.55; 95%CI: 1.82–11.38) and body mass index (BMI) ≥28.0 kg/m^2^ (aOR: 0.16; 95% CI: 0.03–0.91) were significantly associated with sleep disorders in participants aged ≥45 years. Hyperuricemia (aOR: 1.54; 95% CI: 1.09–2.16) and regular exercise (aOR: 0.23; 95% CI: 0.08–0.70) were significantly associated with sleep disorders in the 30–44 years age group.

**Conclusion:**

The mean monthly night flight duration and aging had a synergistic effect on airline pilots’ sleep disorders, implying an aging and work-related mechanistic pathogenesis of sleep disorders in airline pilots that requires additional exploration and intervention.

## 1. Introduction

Previous studies have shown that sleep disorders increase the risk of adverse health outcomes, including obesity, diabetes, cardiovascular diseases, and neurodegenerative diseases (1). Sleep disorders are also responsible for mental health issues such as anxiety, depression, and suicidal ideation (2, 3) and are significantly associated with increased risk of death and accidents (4). Notably, sleep disorders in pilots are a major concern for flight safety. Sleep reduction can make an impact on behavioral alertness and cognitive performance (5), which may reduce pilots’ alertness during flight, resulting in a decline in attention, memory, reaction ability, and driving ability, and even causing flight accidents in severe cases.

Studies conducted on airline pilots have shown a similarly high prevalence of sleep disorders. In a cross-sectional study involving 328 Gulf Cooperation Council commercial airline pilots, 34.1% of participants experienced excessive daytime sleepiness, and 45.1% of individuals reported falling asleep at the controls at least once without first consenting to sleep with their coworkers (6). Another study using the Pittsburgh Sleep Quality Index (PSQI) to measure general sleep quality showed that 38.5% of airline pilots had sleep disorders (7). However, this study was of small sample size, comprising only 41 participants. Most recently, research including 749 aircrew members, 74.1% of whom were pilots, discovered that 45.9% of aircrew members had at least one sleep problem, 24.6% of participants had already involuntarily fallen asleep on board while on duty, and 15.5% of aircrew members had excessive daytime sleepiness (8). However, this study was performed during the COVID-19 pandemic, with many airline pilots not flying at usual times, which might not reflect the impact of flight duty on sleep.

Age was found to be one of the most important factors in predicting sleep health. A previous polysomnographic study of in-flight sleep showed that older aircrew members had longer sleep latencies, more awakenings and arousals, and a smaller number of sleep periods, indicating the impact of age on the quantity and quality of sleep (9). Furthermore, shift work, physical stress at work, current disease, hectic schedules, and gender all contribute to the development of sleep disorders among the general population (10). For airline pilots, their shifting arrangement, demanding work schedules, and time zone changes cause circadian desynchronization and finally lead to the occurrence of sleep problems (11–13). For example, constant stimulation of artificial light and maintaining alertness during the night flight duty may influence circadian clockwork and make them subject to difficulty falling asleep and insomnia after they finish flight duty. However, the question remains as to how night flight duty might impact sleep health. Of note, changes in circadian properties are also associated with aging (14). The molecular circadian clockwork is intricately linked to a number of aging-related signaling pathways (15). As night flight duty itself would disturb the circadian machinery, causing sleep disorders further, it might accelerate sleep disorders among aging airline pilots who are prone to sleep problems. However, no research has been conducted to study the synergistic (interaction) effects of night flight duration and aging on sleep among airline pilots.

This study was designed to investigate the prevalence of sleep disorders among airline pilots and to determine if there is an interaction between age and flight time and sleep disorders. Age-specific correlates that might contribute to the high prevalence of sleep disorders among airline pilots were also examined.

## 2. Materials and methods

### 2.1. Study design and participants

In this cross-sectional study, 1,209 male airline pilots who routinely received a standardized comprehensive physical examination were enrolled from the Shanghai Hospital of Civil Aviation Administration of China, Gubei Branch of Ruijin Hospital Affiliated with the School of Medicine, Shanghai Jiaotong University from July to December, 2021. One participant was excluded for missing data on age, night flight duration, and sleep disorder variables, leaving 1,208 airline pilots with validated information for the final analysis.

The study was approved by the Institutional Review Board (IRB) of Shanghai Hospital of the Civil Aviation Administration of China (Yi Ke Lun Shen No.11[2020]). All participants gave informed consent before taking part in this study.

### 2.2. Assessment of sleep disorders

Sleep disorders were obtained by using PSQI, an instrument that measured the pattern and subjective quality of sleep among airline pilots (7). In general, this 19-item scale measures seven main dimensions of sleep, including subjective sleep quality, sleep efficiency, sleep duration, sleep latency, daytime dysfunction, use of sleep medications, and sleep disturbances. These dimensions of healthy sleep were used to generate a global sleep quality score, ranging from 0 to 21. A higher score indicated poorer sleep quality, and a cut-off value of >5 for the global score denoted poor sleep quality in this study (16).

### 2.3. Data collection

All participants completed a standardized structured questionnaire to evaluate demographics (age, education level, and marital status), lifestyle (smoking status, alcohol use, and exercise), work-related characteristics (flight duration and flight duty in recent year), and physical examination. Smoking status was classified as “never,” “previous,” or “current” according to self-reported information, and regular alcohol use was defined as a self-reported frequency of alcohol use of more than once a week. Regular exercise was defined as engaging in exercise more than three times per week.

The mean monthly flight duration in the 3 years from 2018 to 2020 was obtained by questionnaire and reported by airline pilots themselves. Night flight duration was defined as the length of time of flying a plane between 30 min before sunset to 30 min after sunrise. A long-haul flight was defined as a flight lasting more than 6 h and crossing six time zones. The mean monthly night flight duration and mean monthly long-haul flight duration were both divided into two groups: <30 vs. ≥30 h per month. Similarly, the mean monthly total flight duration was also measured and divided into two groups: <60 h per month versus ≥60 h per month. The flight duty was categorized as student pilot, co-pilot, captain, and pilot instructor.

Height and weight were measured in light clothing by trained public health workers and were used to calculate the body mass index (BMI), the result of body weight divided by height squared (kg/m^2^). According to the criteria of the Working Group on Obesity in China criteria (17), BMI was divided into three groups: normal (<24.0 kg/m^2^), overweight (24.0- < 28.0 kg/m^2^), and obese (≥28.0 kg/m^2^). The data on uric acid (UA) was extracted from the hospital’s electronic medical records system, and hyperuricemia (HUA) was defined as UA ≥ 420 μmol/L (18).

### 2.4. Statistical analysis

Continuous variables were expressed as median (interquartile range, IQR) and categorical variables as numbers and percentages. For continuous variables, the t-test or Wilcoxon Scores test was used, and for categorical variables, the Chi-square test or Fisher’s exact test was used, as appropriate. To assess the linear trend effect of age on sleep disorders, linear models were employed, with age groups treated as the continuous variable. Logistic regression analysis was performed to investigate the interaction between age and mean monthly night flight duration on sleep disorders, including addictive and multiplicative effects, and indicators such as relative excess risk due to interaction (RERI) were used accordingly. Univariate and multivariate logistic regression models were used to calculate the odds ratios (ORs) with 95% confidence intervals (CIs) of mean monthly night flight duration for sleep disorders stratified by age.

We also conducted a sensitivity analysis to investigate the interactive effect of age and mean monthly night flight duration on sleep disorders using a different PSQI global score cutoff value, where a cutoff value of >8 was defined as poor sleep quality in this sensitive analysis. All statistical analysis was performed using Stata 15.0 (Stata Corporation, College Station, TX, USA). A *p*-value of <0.05 indicated statistical significance (two-sided).

## 3. Results

### 3.1. Participant characteristics

Characteristics of aviation pilots with sleep disorders are presented in Table 1. A total of 1,208 participants were included in this study, with a median age of 34 (IQR: 29–39) years. All participants were men, and 573 (47.4%) had a mean monthly night flight duration of ≥30 h in previous three years. Participants with sleep disorders were likely to be in group of 30–44 years, show a lack of physical activity, and have more current smoking, HUA, a longer monthly flight time, and a longer night flight time (*p*s < 0.05).

**Table 1:** Characteristics of aviation pilot by sleep disorders.

Variables	Total (*n* = 1,208)	Sleep disorders		*p-*value
		No (*n* = 693)	Yes (*n* = 515)	
Age groups, yrs				**0.009**
<30	323 (26.7)	216 (29.7)	107 (22.2)	
30–44	726 (60.1)	413 (56.8)	313 (65.1)	
45–62	159 (13.2)	98 (13.4)	61 (12.7)	
Median (IQR)	34(29–39)	34(29–39)	34(30–38)	0.410
Education				0.721
Technical degree	47 (3.9)	28 (3.9)	19 (3.9)	
Bachelor’s degree	1,136 (94.0)	682 (93.8)	454 (94.4)	
Master’s degree	25 (2.1)	17 (2.3)	8 (1.7)	
Marital status				0.064
Single	898 (74.4)	523 (71.9)	375 (78.0)	
Currently married	263 (21.7)	173 (23.8)	90 (18.7)	
Other	47 (3.9)	31 (4.3)	16 (3.3)	
Smoking				**0.015**
Current	351(29.1)	188 (26.0)	162 (33.6)	
Never	667(55.2)	417 (57.4)	251 (52.1)	
Previous	190(15.7)	121 (16.6)	69 (14.3)	
Alcohol use	77(6.4)	39 (5.4)	38 (7.9)	0.077
Regular exercise	50(4.1)	38 (5.2)	12 (2.5)	**0.020**
BMI, kg/m^2^				0.859
<24.0	511(44.2)	309 (44.4)	202 (43.7)	
24.0 – <28.0	529(45.6)	314 (45.1)	215 (46.5)	
≥28.0	118(10.2)	73 (10.5)	45 (9.8)	
HUA	417(34.5)	228 (31.4)	189 (39.3)	**0.005**
Flight duty in the previous year				0.494
Student pilot	91(7.5)	59 (8.1)	32 (6.6)	
Co-pilot	565(46.8)	329 (45.3)	237 (49.2)	
Captain	261(21.6)	157 (21.6)	104 (21.6)	
Pilot instructor	291(24.1)	182 (25.0)	109 (22.6)	
Mean monthly total flight duration >60 h in previous 3 years	1,020(84.4)	600 (82.5)	420 (87.3)	**0.025**
Mean monthly long-haul duration >30 h in previous 3 years	643(53.2)	373 (51.3)	270 (56.0)	0.100
Mean monthly night flight duration >30 h in previous 3 years	573 (47.4)	324 (44.6)	249 (51.7)	**0.014**

IQR, interquartile range; BMI, body mass index; HUA, hyperuricemia. Texts in bold type indicate statistical significance.

### 3.2. Prevalence of sleep disorders

**Figure 1:**
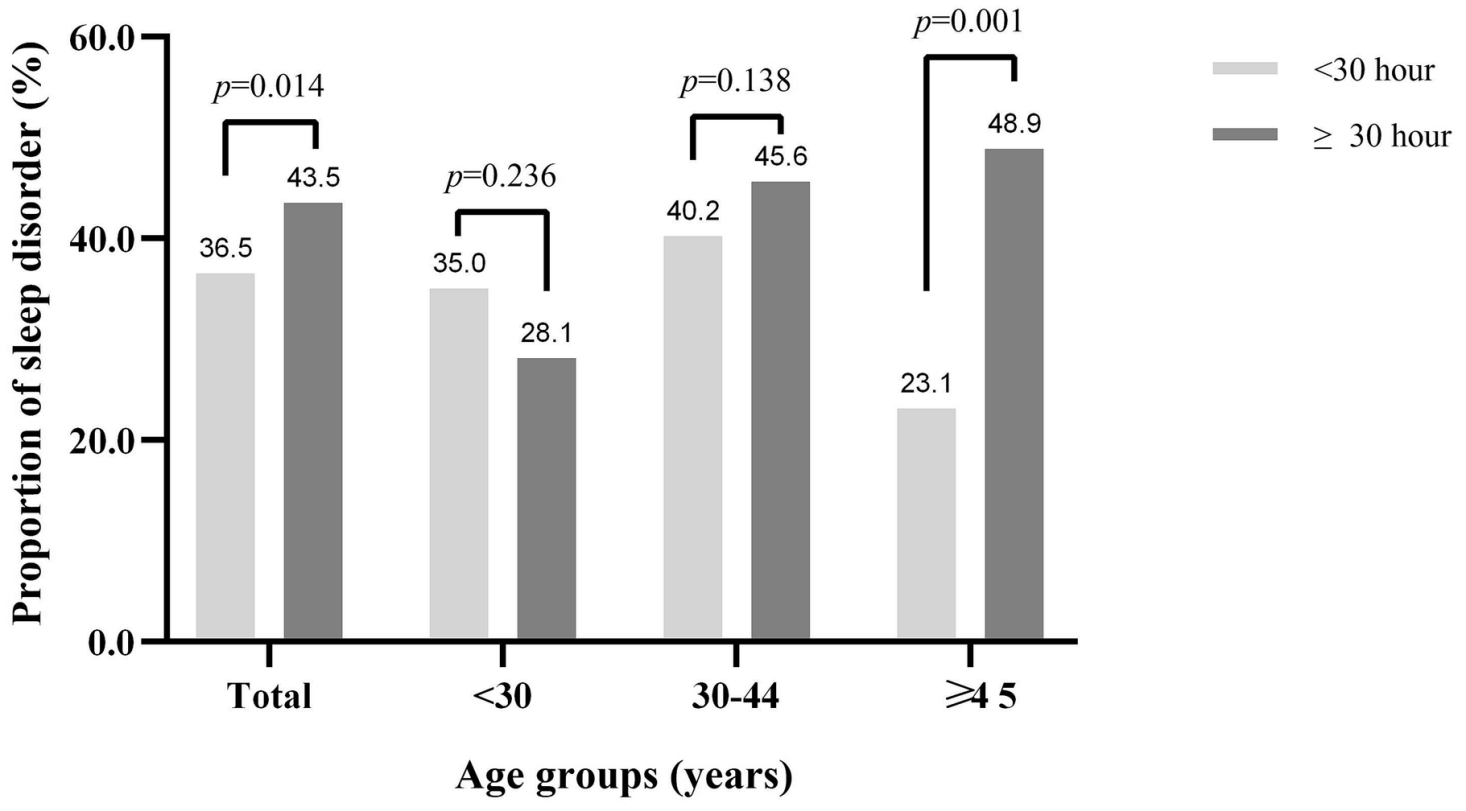
The proportion of sleep disorders by age groups and mean monthly night flight duration in previous 3 years. Test for trend by age for sleep disorders was significant only in the group with night flight durations of ≥30 h in previous 3 years (*p*_trend_ = 0.005).

Overall, 515 (42.6%) participants were detected to have sleep disorders using the PSQI scale. The prevalence of sleep disorders was significantly higher in airline pilots who had night flight durations of ≥30 h than in airline pilots who had a mean monthly night flight duration of <30 h in previous 3 years (43.5 vs. 36.5%, *p* = 0.014). However, inconsistent results were observed across age groups. In the age group ≥45 years, individuals with a longer mean monthly night flight had a significantly higher prevalence of sleep disorders (≥30 h: 48.9 vs. <30 h: 23.1%, *p* = 0.001). However, in the <30 years age group, no significant difference was observed in the prevalence of sleep disorders between different night flight groups (<30 h: 35.0% vs. ≥30 h: 28.1%, *p* = 0.236). Similarly, in the group aged 30–44 years, the prevalence of sleep disorders was 40.2% among airline pilots with night flight durations of <30 h, compared with 45.6% among those who had night flight durations of ≥30 h (*p* = 0.138; Figure 1). Moreover, the prevalence of sleep disorders that increased with age was only observed in individuals with night flight durations of ≥30 h in previous 3 years when stratified by night flight duration (*p*_trend_ = 0.005).

### 3.3. Joint associations of age and mean monthly night flight time with sleep disorders

Given the disparity in the prevalence of sleep disorders among different ages by night flight time, we performed the multivariate logistic regression analysis to examine the joint associations of age and mean monthly night flight time on sleep disorders adjusted for education, marital status, smoking status, regular alcohol use, regular exercise, BMI, HUA, flight duty, mean monthly flight duration in previous 3 years, and mean monthly long voyage duration in previous 3 years (Table 2).

**Table 2:** Joint associations of age and mean monthly night flight duration with sleep disorders.

Age groups	<30 h		≥30 h		aOR (95% CI); *p* for >30 vs. <30 within strata of age group	Measure of effect modification on additive scale: RERI (95% CI)	Measure of effect modification on multiplicative scale: point estimate (95% CI)
	*N* with/without outcome	aOR (95% CI)	*N* with/without outcome	aOR (95% CI)			
<30	85/234	1.00	29/89	0.63 (0.34–1.15) *p* = 0.134	0.63 (0.33–1.22) *p* = 0.173	Reference	Reference
30–44	139/336	1.42(0.88–2.29) *p* = 0.150	192/390	1.75 (1.07–2.86) ***p* = 0.026**	1.17(0.83–1.64) *p* = 0.373	0.70(−1.05–2.46) *p* = 0.433	1.96 (1.01–3.81) ***p* = 0.047**
45–62	24/65	0.61(0.26–1.42) *p* = 0.250	49/94	2.23 (1.07–4.65) ***p* = 0.032**	4.88(1.97–12.11) ***p* = 0.001**	2.00(0.63–3.36) ***p* = 0.004**	5.85 (2.23–15.34) ***p* < 0.001**

RERI, relative excess risk due to interaction. aOR, adjusted odds ratio; aORs are adjusted for education, marital status, smoking status, regular alcohol use, regular exercise, BMI, hyperuricemia, flight duty, mean monthly flight duration in previous 3 years, and mean monthly long voyage duration in previous 3 years. Texts in bold type indicate statistical significance.

In multivariate logistic regression models including the interaction between age and night flight duration on sleep disorders, the adjusted odds ratio (aOR) of interaction term between age and night flight duration on the multiplicative scale was 5.85 (95% CI: 2.23–15.34, *p* < 0.001), and the measure of effect modification on the additive scale of RERI was 2.00 (95% CI: 0.63–3.36, *p* = 0.004), but only for the ≥45 years age group. For the moderately older age group (30–44 years), the hypothesis of the addictive interacted effect between mean monthly night flight duration and moderate older age on sleep disorders should be rejected (*p* = 0.433); while the aOR of interaction terms on the multiplicative scale were 1.96 (95% CI:1.01–3.81, *p* = 0.047), suggesting that the hypothesis of multiplicative effect between age and night flight duration on sleep disorders was accepted. These results show an aggravating effect of night flight duration on sleep health as the age of airline pilots increases (Table 2).

### 3.4. Age-specific correlates of sleep disorders

Table 3 presents the results of multivariate logistic regression analysis stratified by age groups. The result indicates that longer night flight duration (aOR = 4.55, 95% CI: 1.82–11.38, *p* = 0.001) and BMI of ≥28.0 kg/m^2^ (aOR = 0.16, 95% CI:0.03–0.91, *p* = 0.039) were significantly associated with sleep disorders in the ≥45 years age group. Among the 30–44 years age group, sleep disorders were positively associated with HUA (aOR = 1.54, 95%CI: 1.09–2.16, *p* = 0.013) but negatively associated with regular exercise (aOR = 0.23, 1 95%CI: 0.08–0.70, *p* = 0.010).

**Table 3:** Factors associated with sleep disorders by age groups.

Variables	Aged <30 years		Aged 30–44 years		Aged ≥45 years	
	aOR (95% CI)	*p*-value	aOR (95% CI)	*p*-value	aOR (95% CI)	*p*-value
Age, year	1.04(0.87–1.24)	0.652	0.98(0.93–1.04)	0.547	1.04(0.95–1.14)	0.405
Education						
Technical degree	1.00		1.00		1.00	
Bachelor’s degree	-	-	-	-	1.09(0.42–2.82)	0.857
Master’s degree	2.60(0.15–44.38)	0.509	0.86(0.29–2.53)	0.780	0.31(0.03–3.28)	0.330
Marital status						
Single	1.00		1.00		1.00	
Currently married	0.78(0.44–1.41)	0.412	0.77(0.43–1.36)	0.366	-	-
Others	0.97(0.08–12.21)	0.980	0.59(0.29–1.20)	0.145	0.61(0.10–3.73)	0.596
Smoking						
Current	1.00		1.00		1.00	
Never	0.65(0.36–1.17)	0.153	0.79(0.55–1.12)	0.181	1.35(0.52–3.50)	0.541
Previous	0.46(0.16–1.30)	0.142	0.65(0.40–1.06)	0.085	1.28(0.50–3.26)	0.604
Alcohol use in previous 3 years	2.36(0.45–12.26)	0.307	1.22(0.66–2.28)	0.522	1.21(0.36–4.09)	0.764
Regular exercise	1.84(0.47–7.14)	0.378	0.23(0.08–0.70)	**0.010**	0.34(0.08–1.42)	0.139
BMI, kg/m^2^						
<24.0	1.00		1.00		1.00	
24.0 – <28.0	0.90(0.53–1.54)	0.706	0.99(0.70–1.38)	0.937	0.61(0.28–1.30)	0.199
≥28.0	0.79(0.32–1.95)	0.607	0.83(0.49–1.40)	0.475	0.16(0.03–0.91)	**0.039**
HUA	1.27(0.75–2.14)	0.372	1.54(1.09–2.16)	**0.013**	1.94(0.87–4.34)	0.106
Flight duty in the previous year						
Student pilot	1.00		1.00		1.00	
Co-pilot	0.81(0.42–1.57)	0.533	0.86(0.11–6.81)	0.884	-	-
Captain	-	-	0.66(0.08–5.46)	0.703	-	-
Pilot instructor	-	-	0.61(0.07–5.08)	0.645	1.07(0.47–2.45)	0.871
Mean monthly total flight duration >60 h in previous 3 years	1.78(0.93–3.40)	0.080	1.02(0.50–2.10)	0.952	1.07(0.25–4.57)	0.925
Mean monthly long-haul duration >30 h in previous 3 years	0.56(0.29–1.09)	0.086	1.20(0.83–1.72)	0.332	0.70(0.25–1.95)	0.497
Mean monthly night flight duration>30 h in previous 3 years	0.63(0.33–1.22)	0.173	1.17(0.83–1.64)	0.369	4.55(1.82–11.38)	**0.001**

BMI, body mass index; HUA, hyperuricemia. Texts in bold type indicate statistical significance.

### 3.5. Sensitive analysis

In sensitive analysis, the interaction term of age and mean monthly night flight duration on sleep disorders lost its statistical significance using the PSQI global score ≥ 8 as a cutoff value for sleep disorders. However, in a multivariate logistic regression model without interaction terms, mean monthly night flight duration was significantly associated with sleep disorders (aOR = 2.12, 95% CI: 1.35–3.33). In an age-specific regression model, the mean monthly night flight duration was significant in the 30–44 years age group (aOR = 2.19, 95% CI: 1.27–3.78) and the ≥45 years age group (aOR = 5.63, 95% CI: 1.06–29.94; Supplementary Table S1).

## 4. Discussion

In this cross-sectional study comprising 1,208 male airline pilots, we found a relatively high prevalence of sleep disorders among airline pilots. We identified a positive association of age and night flight duration with sleep disorders in airline pilots. Specifically, the mean monthly night flight duration of ≥30 h in previous 3 years was significantly associated with a higher risk of sleep disorders in pilots aged ≥45 years. Moreover, age-specific factors including clinical and lifestyle characteristics were also observed. To our knowledge, this is the largest study exploring the prevalence of sleep disorders by PSQI scale and the association of age and night flight duration with it among airline pilots in Asia.

Sleep problems are common in shift workers, such as healthcare professionals (39.2%) (19), night shift autoworkers (56.2%) (20), and firefighters (59.3%) (21), and their prevalence is relatively higher than the general population (16.6–35.9%) (22–24). In this study, we observed a higher prevalence of sleep disorders in professional airline pilots, mirroring the result obtained from national commercial pilots in Saudi Arabia (6, 7). Sleep problems are one of the problems concerning airline pilots since lack of good sleep causes fatigue and decrements in performance and consequently poses a threat to flight safety. Previous studies used wrist-based actigraphy to measure or estimate individuals’ sleep amounts during flight duty, based on which rest schemes were made to solve sleep problems, but some findings countered the current literature and recommendations (25, 26). Therefore, more large population-based studies were needed to provide evidence of sleep and fatigue management.

It has been hypothesized that age-related changes in the human circadian pacemaker and sleep homeostatic mechanisms play a pivotal role in the hallmarks of age-related changes in sleep (27). Both subjective and objective measures of sleep indicate that sleep patterns and characteristics change with increasing age, and the prevalence of sleep disorders is higher among older adults (28, 29). In this study, we found an increasing prevalence of sleep disorders by age, in agreement with previous studies.

Although the flying duration and flight duty times of each pilot are restricted and proper rest periods are mandated by China Civil Aviation Regulations to guarantee flight safety, sleep disorders remain a common problem among airline pilots. In this study, only the mean monthly night flight duration was significantly associated with sleep disorders. Notably, our results show an interactive effect of mean monthly night flight duration and age on sleep, which suggests that duration of night flight and age are not isolated factors for sleep disorders. Among airline pilots aged ≥45 years, longer night flight duration increases the risk of sleep disorders, which suggests a complex impact of aging on circadian rhythms. Although how aging affects the circadian clock is still unclear, aging could weaken the circadian clock, disrupt sleep–wake cycles, reduce the ability of peripheral organs to coordinate circadian rhythms, and change the circadian clock output at the molecular level (15). The long night flight time could cause more sleep problems in senior pilots since night flights have been linked to circadian rhythm disturbance. The findings in our study indicate the necessity for age-specific night flight time restrictions among airline pilots.

Consistent with other findings that short night sleep duration is associated with a higher risk of HUA (30), our data also shows a positive relationship between HUA and sleep disorders, but only among airline pilots aged 30–44 years. Moreover, we could not determine whether high levels of UA lead to sleep disorders in this study. In this study, we observed that regular exercise was negatively associated with sleep disorders among middle-aged and older adult airline pilots, although the association was not significant for those aged ≥45 years, which suggests that regular exercise habits might benefit sleep. In contrast to a previous study, which showed a negative association between obesity and sleep health (31), we found that obesity was positively associated with sleep health among airline pilots aged over 45 years, which needs further investigation. It has been reported that men’s sleep is more prone to be influenced by aging than women’s (32). However, limited by the gender of the participants, we are unable to explore this.

Sleep disturbance not only contributes to biological aging, cognitive aging, and depression (33) but is also associated with cardiovascular disease and related harmful outcomes (34, 35). A meta-analysis, which comprised 61 original studies from 71 different populations, has revealed a significant negative effect of sleep restriction on cognitive processing across cognitive domains (36). Moreover, sleep disturbances are associated with faster chronic disease accumulation, which points towards the importance of early detection and treatment of sleep disturbances in older adults (37). Our findings reveal the necessity of sleep management as a possible strategy to reduce chronic multimorbidity and guarantee flight safety for airline pilots. An age-specific arrangement of restricted duty time and scientific rest, which would be helpful to pilots’ health and extend their careers, would be beneficial for commercial airline companies in the long term.

This study has several limitations. First, we conducted a cross-sectional study; therefore, we could not confirm the causation between the development of sleep disorders and associated factors of interest, such as HUA and regular exercise. Nevertheless, it was unlikely to influence our finding of the synergistic effect of age and mean monthly night flight time on sleep disorders. Second, sleep disorders were determined by self-reported questionnaires but not polysomnography. Self-report data are prone to information bias, as participants might exaggerate or understate their sleep problems. However, as participants completed the questionnaire individually without any intervention from their colleagues or healthcare workers, the results pooled from the questionnaires should reflect the real sleep problems of airline pilots at the population level. Moreover, a standard PSG is usually conducted in a sleep lab with a certified sleep technician present throughout the study, which is time-consuming and complex for a population-based epidemiological study. A recent study suggested that subjective sleep quality was significantly associated with changes in cortisol levels that reflect the circadian rhythm of the Hypothalamic–Pituitary–Adrenal (HPA) axis (38). Therefore, the PSQI would be a proper measure of pilots’ sleep issues, especially in large-scale population studies. Third, all participants were recruited from one commercial airline company, which may limit the generalizability of the results in view of different airline arrangements and rest schemes in airline companies. However, we only examine the association of mean monthly night flight duration with sleep disorders in this study, Therefore, our observed association should not be affected. Finally, the results may not generalize to female airline pilots because they are limited to male pilots.

## 5. Conclusion

We found that the mean monthly night flight time and aging had a synergistic effect on airline pilots’ sleep disorders, with those who were ≥ 45 years old and had longer night flight durations being at higher risk. We also discovered that the overall prevalence of sleep disorders increased with age. However, no such occurrence was observed among those whose average monthly night flying duration was <30 h, emphasizing the importance of proper night flight duty assignment in consideration of age. Extra consideration should be given to the negative effects of metabolic changes on sleep disorders. Additionally, regular exercise may help middle-aged airline pilots sleep better.

## Data availability statement

The original contributions presented in the study are included in the article/Supplementary material, further inquiries can be directed to the corresponding author.

## Ethics statement

The studies involving humans were approved by the Institutional Review Board (IRB) of Shanghai Hospital of the Civil Aviation Administration of China. The studies were conducted in accordance with the local legislation and institutional requirements. The participants provided their written informed consent to participate in this study.

## Author contributions

RS contributed to the data analysis and interpretation and drafted the manuscript. LF and FW contributed to the conception and design of the work and data collection. LF and WX critically reviewed the manuscript. LF generally supervised the study. All authors contributed to the article and approved the submittedversion.

## Funding

This study was supported by the Security Improving Project of the Civil Aviation Administration of China - The establishment of comprehensive support system for flight safety based on airline pilot health management (the grant number was not applicable).

## Conflict of interest

The authors declare that the research was conducted in the absence of any commercial or financial relationships that could be construed as a potential conflict of interest.

## Publisher’s note

All claims expressed in this article are solely those of the authors and do not necessarily represent those of their affiliated organizations, or those of the publisher, the editors and the reviewers. Any product that may be evaluated in this article, or claim that may be made by its manufacturer, is not guaranteed or endorsed by the publisher.
