# Supplementary material for: Association of age and night flight duration with sleep disorders among Chinese airline pilots
Source: Front Public Health. 2023 Sep 7;11:1217005. doi: 10.3389/fpubh.2023.1217005 (PMC10513407; doi:10.3389/fpubh.2023.1217005)
Supplement: Supplementary file 1 [file Table_1.docx]

Supplementary Material

Association of Age and Night Flight Duration with Sleep Disorders among Chinese Airline Pilots

**Ruizi Shi^1^, Fang Wang^2^, Wanying Xu^1^, Li Fu^2,^ ***

^1^ Shanghai Institute of Aviation Medicine, Ruijin Hospital Affiliated to School of Medicine, Shanghai Jiao Tong University, Shanghai, China;

^2^ Shanghai Hospital of Civil Aviation Administration of China, Gubei Branch of Ruijin Hospital Affiliated to School of Medicine, Shanghai Jiaotong University, Shanghai, China.

*** Correspondence**

Li Fu, associate chief physician, Shanghai Hospital of Civil Aviation Administration of China, Gubei Branch of Ruijin Hospital Affiliated to Shanghai Jiaotong University School of Medicine, No. 398 Hongbaoshi Road, Shanghai, 200336, China.

Email: fu.li@rjhgb.com

**Table S1 The association of mean monthly night flight duration with sleep disorder categorized by age groups**

| Age groups | Model 1 | | |  | Model 2 | | |
| --- | --- | --- | --- | --- | --- | --- | --- |
|  | <30 hour | ≥30 hour | *p* value |  | <30 hour | ≥30 hour | *p* value |
| <30 | 1.00 | 1.66 (0.68-4.08) | 0.267 |  | 1.00 | 0.86 (0.27-2.71) | 0.798 |
| 30-44 | 1.00 | 2.16 (1.34-3.49) | **0.002** |  | 1.00 | 2.19 (1.27-3.78) | **0.005** |
| ≥45 | 1.00 | 5.63 (1.23-25.71) | **0.026** |  | 1.00 | 5.63 (1.06-29.94) | **0.043** |
| Total* | 1.00 | 2.34 (1.57-3.47) | **<0.001** |  | 1.00 | 2.12 (1.35-3.33) | **0.001** |

*Age was included in model 1 and model 2 as categorical variable. Texts in bold type indicate statistical significance

Model 1 adjusted for age (as continues variable); 2 adjusted for age (as continues variable), education, marital status, smoking status, regular alcohol use, regular exercise, BMI, hyperuricemia, flight duty, mean monthly flight duration in previous 3 years and mean monthly long-haul duration in previous 3 years.
